# Methods for co‐designing health innovations with older adults: A rapid review

**DOI:** 10.1111/ajag.70065

**Published:** 2025-07-16

**Authors:** Léa Blouin Gagnon, Samuel Turcotte, Roxane DeBroux‐Leduc, Philippe Gauthier, Nathalie Bier

**Affiliations:** ^1^ Centre de Recherche de l'Institut Universitaire de Gériatrie de Montréal (CRIUGM) – Centre intégré universitaire de santé et services sociaux (CIUSSS) du Centre‐Sud‐de‐l'île‐de‐Montréal Montréal Québec Canada; ^2^ School of Psychology Université Laval Québec Canada; ^3^ Centre interdisciplinaire de recherche en réadaptation et intégration sociale (Cirris) du CIUSSS de la Capitale‐Nationale Québec Canada; ^4^ School of Rehabilitation Sciences Université Laval Québec Canada; ^5^ School of Rehabilitation Université de Montréal Montréal Québec Canada; ^6^ School of Design Université de Montréal Montréal Québec Canada

**Keywords:** ageing, community‐based participatory research, review

## Abstract

**Objective:**

This paper reviews the methods used in research for co‐designing health innovations with and for older adults.

**Methods:**

A rapid review was performed following the Cochrane Rapid Review Methods Group recommendations and reported using the PRISMA statement. A keyword search was conducted in CINAHL, PsychINFO and Medline databases.

**Results:**

Selection criteria yielded 13 peer‐reviewed articles that reported on a total of 33 different co‐design activities. Discussions and individual interviews were the most frequently reported activities in the selected articles and were used at each stage of the co‐design process.

**Conclusion:**

To avoid the appearance of tokenism and to promote transparency, researchers need to provide more details about the involvement of older adults in the co‐design process and the co‐design activities performed. Health and design scientists should strive towards a meaningful involvement of older adults throughout the co‐designing process and need to develop knowledge about the added value of co‐design to support the use of such methods.

**Practice impact**

Researchers wishing to conduct a co‐design study with older adults could use the present review to learn about different co‐design activities, think creatively about how to adapt their activities, or gain inspiration from other studies.

## 1 INTRODUCTION

New health programs and medical innovations can only be developed once knowledge about the specific needs and challenges of different populations, and the characteristics of their life context has been collected. With the ageing of the global population,^1^ finding ways to better assess these is becoming critical. This population is more likely to experience health issues such as hearing and vision impairment, physical pain, diabetes, depression and neurocognitive disorders.^1^ Differences in cultural, social, economic and environmental backgrounds also give each individual a unique perspective on life and the way they wish to live it. Researchers, innovators and designers should identify and take into account, without bias, the differing needs, issues and worldviews of older adults. It is therefore crucial to adapt research approaches and to provide researchers with tools to comprehensively grasp the various characteristics that define older adults.

One way of meaningfully addressing the health‐related demands and concerns of older adults is to actively include them in the process of designing solutions. The experimentation and dissemination of human‐centred design (HCD) in many professional fields has given rise to a whole new perspective on how older adults and other stakeholders, such as caregivers and health‐care professionals, can participate in improving their living and work environments. The tenets of HCD have contributed to experts' interests in working together with users, rather than considering them as subjects. HCD has shown how users can bring their perspectives and ideas to the innovation process and actively contribute to finding answers to their common problems.^2^ According to Don Norman, HCD ‘ensures that the designs match the needs and capabilities of the people for whom they are intended’.^3^ ^, p. 9^ Methods developed in the context of HCD are increasingly being used in health research. For example, one study used HCD to develop an innovation that assists primary care providers and patients living with chronic kidney disease in identifying and treating hypertension.^4^ The authors reported that the HCD process led to a better comprehension of the users' needs and a more optimal design of the innovation. In another study, HCD methods were used to co‐develop a digital health technology that would help to better engage patients with heart failure in self‐care behaviours.^5^ Human‐centred design encompasses a wide range of methods, including co‐design, that lead to the design of services or technologies centred on user needs.^6^

Co‐design has only recently become a popular tool in health research, despite its longer history.^7^ Both creativity and design are considered integral parts of co‐design.^8^ As mentioned by Holl and Cruysen: ‘Design is a structured process that transforms creative ideas into concrete products, services and systems, and links creativity to innovation’.^8^ ^, p. 3^ The prefix ‘co’ underlines the fact that the process is carried out collaboratively between various actors from different spheres (e.g. citizens, researchers, representatives of community organisations or private companies).^9^

There are different conceptual frameworks outlining the design process that indicate at what stages and how collaboration with the stakeholders of an innovation can take place. These include the Double Diamond method, a visual representation of the steps (discover, define, develop and deliver) undertaken during a design process,^10^ the Experience‐based co‐design approach, a ‘user‐focused design process with the goal of making user experience accessible to the designers, to enable them to focus on designing experiences rather than services’^11^ ^, p. 308^ and the Design Thinking approach.^12^ Brown's Design Thinking will be used throughout this article to provide more structure to the analysis of the included studies.^12^ Although abstract, this model offers the advantage of providing a broad understanding of the design process that does not prescribe when and how collaboration should occur, making it an interesting analytical tool. According to Brown,^12^ a co‐design process should involve the following stages: (1) empathising and defining (clarifying the problem), (2) ideating (generating ideas and solutions to solve the problem), (3) prototyping (creating an innovative prototype to solve the problem) and (4) testing (evaluating the prototype). This highly iterative process often requires moving back and forth between the different stages several times before the final product is ready for implementation.^13^

Researchers conducting a study may want to involve participants from different backgrounds in the iterative process. They might wish to involve their collaborators in a wide range of design activities (e.g. art installations, persona‐scenarios and storyboarding) during the four stages of the iterative process. Co‐design is meant to engage experts, designers and potential stakeholders in thinking creatively about solutions to certain issues while identifying those that are most relevant to them. However, methodological literature on the use of co‐design in research activities and peer‐reviewed guidelines on creating health innovations with and for older adults remains scarce.^9^ Some reviews have previously synthesised which co‐design activities have been used (and why) when working with or for older adults, while also touching on the involvement of older adults.^14^, ^15^ Merkel et al.^14^ focused on categorising the different types of participatory design methods and how they are implemented in the field of gerontotechnology. Fisher, Peine and Östlund^15^ aimed at understanding the nature and the implications of involving older adults in the design of technologies. However, none of these reviews have precisely described what the co‐design activities consist of. Considering co‐design's growing popularity in health research, greater insight is needed into what could be reliable techniques for conducting such processes with older adults. As a result, this study aimed to briefly synthesise knowledge on the methods used for co‐designing health innovations with and for older adults as reported in research papers. The key question is: What are the methods currently used in research when co‐designing health innovations with and for older adults? The present review focuses on describing, in detail, co‐design activities in research, which encompasses any meeting between members of a research team and various stakeholders. Relevant systematic reviews have found that using multiple co‐design activities increases the opportunities for participants to actively contribute to the co‐design process, since different activities seek out different skills (e.g. visual, physical or social skills).^16^, ^17^ Researchers wishing to conduct a co‐design study with older adults could use the present review to learn about different co‐design activities, think creatively about how to adapt their activities or gain inspiration from other studies. For each study reviewed, the key question was divided into four sub‐questions:

1. What was the purpose of the co‐design process?
2. In which stage were older adults involved in the co‐design processes according to Brown's Design Thinking framework?
3. Which activities were used and how they were performed when co‐designing with and for older adults?
4. Which activities were involved at each stage of the design thinking framework suggested by Brown?^12^

## 2 METHODS

### 2.1 Research design

A rapid review was conducted to address the research questions. According to Grant^18^ ^, p. 5^ a rapid review consists of ‘assess[ing] what is already known about […] a practice issue, by using […] methods to search and appraise existing research’. While systematic reviews are exhaustive and therefore longer, rapid reviews are an alternative in which information is provided in a synthesised and time‐efficient way.^19^ Resorting to this type of review was deemed necessary because the project had to be carried out within a 2‐month time frame to inform an ongoing research project using co‐design with and for older adults.

This review was performed according to the Cochrane Rapid Review Methods Group recommendations^20^ and reported using the Preferred Reporting Items for Systematic reviews and Meta‐Analyses (PRISMA) statement.^21^ The steps followed in the rapid review process involved defining the research question, establishing the eligibility criteria, conducting a literature search, selecting studies for review, extracting data from the studies and synthesising the data.^20^

### 2.2 Eligibility criteria

The first inclusion criterion was that the publication does not predate 2010, considering the rapid review's short time frame; this was also reasonable giving the scarcity of published literature on co‐design with older adults in health research prior to that year. The journal article needed to be published in English or French and had to explain the co‐design process in detail (e.g. activity, equipment and location). The study had to be conducted in the field of health research, which includes—but is not limited to—health promotion, prevention, rehabilitation, organisational health, physical, mental or social health research. The health innovation(s) mentioned had to be co‐designed to serve older adults (aged 55 or older). While the literature often defines older adults as being aged 60–65 and older, no consensus exists. In the context of our study, setting the age criterion at 55 years old would help include more relevant articles and a wider variety of people. Indeed, people ageing with specific health related needs or disabilities might report ageing more quickly or feel like being ‘old’ from a younger age.^22^ Older adults may or may not have been involved at each stage of the iterative co‐design process. Participation from other stakeholders such as family members, health‐care professionals or community members still qualifies as co‐design. In terms of exclusion criteria, the decision was made to focus on journal articles, which excluded grey literature, conference abstracts and poster abstracts. Literature reviews were also excluded, insofar as the details about methods and activities that were of interest may not be covered.

### 2.3 Databases and search strategy

Advanced keyword searches were conducted in the CINAHL, PsychINFO and Medline databases. It was decided to limit searches to three databases, as recommended for rapid reviews by Garrity et al.^20^ The choice of these databases was made with the help of a librarian specialised in health research. The searches were carried out between January and February 2021. A search strategy was developed and validated with the same librarian. Key terms used to perform the search stemmed mainly from the following four concepts: Co‐design, Health, Older Adults and Methods. The search entry for the three databases is available in Appendix S1. The key terms were searched respectively in the Abstract, Keyword/Abstract and Title/Abstract fields of the three databases.

### 2.4 Screening process and study selection

**FIGURE 1:**
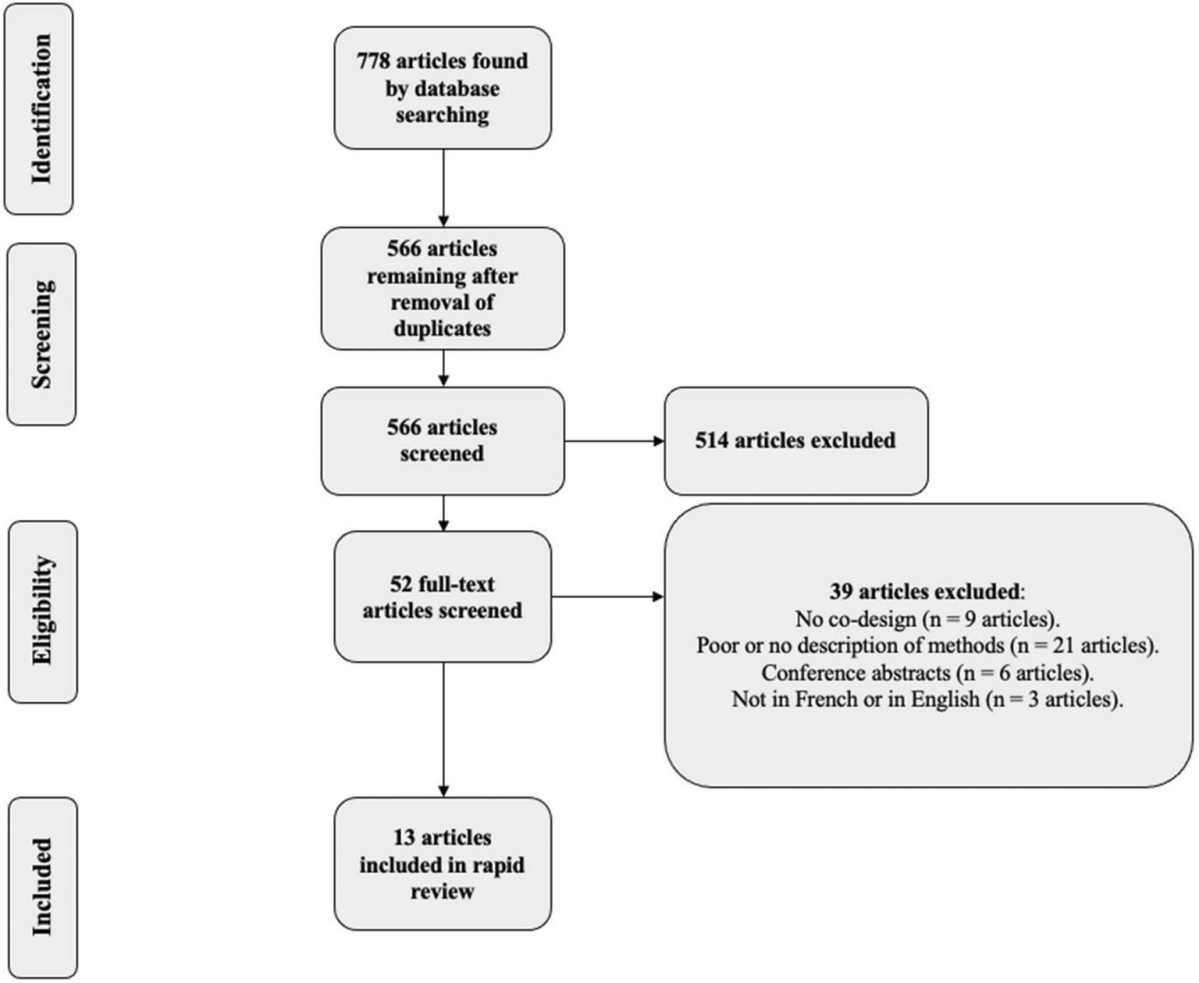
Study selection flowchart.

After completing the literature search, 778 documents were uploaded in Zotero (version 5), a reference management software. From there, duplicates were removed, leaving 566 documents. As part of the selection process, after verifying the inclusion and exclusion criteria validity by screening 30 randomly chosen articles (the first 30 articles in alphabetical order), criteria were applied to screen the titles and abstracts of the 566 papers. Three co‐authors (LBG., ST., RDL) participated independently in this screening test to single out articles that corresponded to the criteria. Since there were no differences of opinion between co‐authors, only one of them (LBG) completed the rest of the initial selection process. Only 52 articles remained after this first phase of eligibility screening. The second part of the selection process involved applying the criteria to the full text of these articles. First, a selection test was performed on 10 randomly chosen articles (the first 10 articles in alphabetical order) by the same three co‐authors independently, and since no differences of opinion emerged, the second part of the selection was completed by one of the co‐authors (LBG). This narrowed down the number of studies to 13. The following flow chart was created according to the PRISMA statement^21^ See Figure 1.

### 2.5 Data extraction process

An extraction table was created in Excel through an iterative process involving four co‐authors (LBG, ST, RDL, NB), leading to the identification of the extraction table content and the definition of each category. Thus, when available, the following information was extracted: year of publication, country of origin, aim of study, purpose of co‐design process, level of collaboration of older adults in the co‐design process and results of study. In addition, for each of the activities carried out within the co‐design process, the following information was extracted where available: characteristics of people involved in the co‐design activity (e.g. age, biological sex, occupation, level of education), nature of co‐design activity, aim of co‐design activity, length of activity and participants' compensation for their contribution. Data from the 13 articles were extracted by one of the co‐authors (LBG). A second author checked for correctness and completeness of extracted data against the included articles.

### 2.6 Synthesis of results

The data were synthesised narratively, including tables illustrating the extracted data from the studies as recommended by the Cochrane Rapid Review Methods Group Guide.^20^ Narrative syntheses include synthesising the data to further understand and explain the findings. The data were summarised both deductively, using Brown's Design Thinking framework^12^ and inductively for other extracted information (e.g. length of co‐design activity). More specifically, similarities and differences between the studies were investigated to reveal the presence of main tendencies that could inform co‐design practices. Descriptive statistics were also performed where relevant (e.g. ranges, mean and frequencies) to further interpret the data. Each of the co‐design activities identified were categorised according to Brown's Design Thinking framework.^12^

## 3 RESULTS

### 3.1 Study characteristics and purposes of the co‐design processes

The studies reported in the 13 articles selected for analysis were conducted in different countries around the world. Eleven articles of 13 were published between 2016 and 2020; the other two were published in 2010 and 2012. The purposes of the co‐design processes according to each study were divided into five categories: (1) understanding the needs, lived experiences and problems of older adults; (2) thinking about and developing possible solutions or services; (3) testing solutions or services; (4) supporting the adoption of technologies; and (5) creating a user network. Every study specified that understanding the needs, lived experiences and problems of older adults was the purpose of adopting a co‐designing approach. In addition, every study, except for one,^23^ stated that the co‐design process is intended to think about and develop possible solutions or services. The exception, Lu et al.,^23^ might be explained by the fact that the aim of that study was to determine the contribution of a co‐creation process to the adoption of technologies. Therefore, the purpose of the co‐design for that study was focussed on supporting the adoption of technologies. The study by Righi et al.^24^ was the only one in which the co‐design process was designed to create a user network. Other characteristics such as the description of participants, the co‐design activities and the results of the studies are presented in Table 1.

**TABLE 1:** Characteristics of included articles.

Author, year (country)	Aim of study	Participants	Purpose of co‐design	Co‐design activities	Study results
Brett et al. (2019) (Australia)^25^	The Ageing Well project was aimed at evaluating the level of social participation and quality of life of aged care clients in their community. It also gained insight into stakeholder perspectives for use in enhancing care planning	23 participants from culturally and linguistically diverse backgrounds, 80 years and older, from regional areas	Understand the needs, lived experiences and problems of older adults Think about and develop possible solutions or services	Focus groups Stakeholder forums	The forums enabled stakeholders (policy‐makers, clients and care coordinators) who rarely meet to reflect on their aged care experiences. They were able to identify relevant needs and priorities for improvement of aged care services, and to devise strategies to address these needs
Brookfield et al. (2020) (UK)^26^	Promoting engagement of older adults in the design of supportive environments to facilitate healthy aging by understanding the potential value of 8 engagement techniques (activities)	93 participants; mostly community residing older adults, stroke survivors and people living with major neurocognitive disorders	Understanding the needs, lived experiences and problems of older adults Thinking about and developing possible solutions or services Testing co‐design methods	Photovoice Model‐making Walking interviews Participatory mapping exercises Drawing Design fairs Photo‐elicitation Talking mats Focus groups	The 8 techniques used to engage older adults indicate that there are multiple ways to involve this age group in designing age‐friendly homes and neighbourhoods
Dorant and Krieger (2017) (The Netherlands)^27^	Coordinating and professionalising family caregiver support initiatives with Vade Mecum, an innovative support concept for older adults' family caregivers. The project is aimed at sustaining wellbeing, social inclusion and health equity	16 participants: physicians (*n* = 2), case managers (*n* = 2), social workers (*n* = 2), physiotherapist (*n* = 1), logotherapist (*n* = 1), nurses (*n* = 4), external research facilitators (*n* = 2), nurse teacher (*n* = 1), pastor working with the geriatric department (*n* = 1). The authors specified that family caregivers were not yet involved in this initial phase of the project	Understanding the needs, lived experiences and problems of older adults Thinking about and developing possible solutions or services	Brainstorming Discussion Focus groups	During this first phase of the project, a high degree of participation was achieved. The participants felt empowered, new knowledge was co‐created, and research questions were formulated. Solutions to the research problem were brought forward and suitable data collection methods were agreed on
Fang et al. (2016) (Canada)^28^	Participatory community mapping workshops were used to see how the concept of ‘sense‐of‐place’ could be translated into concrete solutions for older adults in a context of designing and developing home environments	38 older adults (aged 60 and over, from diverse cultural backgrounds, English and/or Cantonese speakers) and 16 local service providers	Understanding the needs, lived experiences and problems of older adults Thinking about and developing possible solutions or services	Experiential group walks Participatory mapping exercises	Participatory community mapping workshops enabled the identification of actionable solutions to enhance the feeling of ‘sense‐of‐place’ and showed how these solutions could be established in spaces. Participatory community mapping workshops also helped identify health and wellbeing needs, and opportunities for social participants to overcome cross‐cultural challenges
Hoffman et al. (2019) (USA)^29^	Engaging older adults and their families in the development and testing of an interactive website aimed at helping families to make long‐term health‐care decisions	Patients, health‐care professionals, and family members who were able to read, write, and understand English	Understanding the needs, lived experiences and problems of older adults Thinking about and developing possible solutions or services Testing solutions or services	Persona‐scenarios Storyboarding Think aloud sessions Interviews Focus groups	Production of an interactive website providing information and decision‐making support tailored to older adults and their local and long‐distance caregivers
Kelemen et al. (2018) (UK)^30^	The ‘A picture of Health’ research project aimed at co‐designing research questions that were in line with the experiences of the participants; especially participants who found themselves on the margins of society. The project was performed using a Cultural Animation method	24 participants (4 academics, 5 retired health professionals, 15 community members). Participants' age ranged from 25 to 75 years. 80% of participants were women and aged 60 and over	Understanding the needs, lived experiences and problems of older adults Thinking about and developing possible solutions or services	Creating art installations Creation of a three‐dimensional Facebook page and a fictional Tweet Creation and presentation a human tableau	Findings suggest that Cultural Animation can unearth hidden assets in the community, increase human connectivity, rethink the meaning of health, and co‐create new health indicators grounded in day‐to‐day experience. It enables people to think of their communities in a more positive way and helps to consider what can be developed on the basis of structures already in place
Leask et al. (2017) (UK)^31^	Co‐creating a public health intervention to reduce sedentary behaviour in older adults	11 community‐residing older adult volunteers (aged 65 and over; age range = 66–82; mean age = 74; 5 men). 4 university researchers	Understanding the needs, lived experiences and problems of older adults Thinking about and developing possible solutions or services	Discussions Fieldwork tasks	Development of the ‘Are you up for it?’ intervention aimed at reducing sedentary behaviour and containing key elements delivered through a mode congruent with older adults' daily lives
Lu et al. (2017) (The Netherlands)^23^	Determining the contribution of 3 ideation‐stage co‐creation processes and the adoption of technologies aimed at promoting physical activity in older adults	Older adult participants	Understanding the needs, lived experiences and problems of older adults Supporting the adoption of technologies	Experience mapping Persona‐scenarios Discussion	The 3 ideation‐stage co‐creation processes partly support the adoption of technologies
O'Brien et al. (2016) (UK)^32^	Developing a complex web‐based health intervention aimed at promoting the health and wellbeing of retired people	The participants were members of the research team (research facilitators and scribes), adults aged 55 and over who were potential intervention users, health and social care professionals from the voluntary sector and public health organisations	Understanding the needs, lived experiences and problems of older adults Thinking about and developing possible solutions or services Testing solutions or services	Persona‐scenarios Experience mapping Storyboarding Profile question making Cognitive walkthrough	The final output is a functional prototype for LEAP, which is ready for formal testing to ensure the effectiveness of the intervention
Punnaraj et al. (2010) (Thailand)^33^	Determining whether a Community Capacity Model is appropriate for providing care for the elderly by gathering data about their needs via different participatory methods, and by involving them in the development of the community health‐care model	140 older adults (aged 60 and over; mean age = 71.2; 81 females), 20 family members of older adults, 11 community leaders, 5 nurses, 10 health‐care volunteers and 5 local government officials	Understanding the needs, lived experiences and problems of older adults Thinking about and developing possible solutions or services	Interviews Focus groups Brainstorming	The proposed Community Capacity Model was found to be a workable partnership model based on the resources and capabilities of the community. It was also found to reinforce community involvement within the social and cultural context by focusing on the role of family and community networks in providing care, with support from the local health‐care unit and the administration
Righi et al. (2017) (Spain)^24^	Finding ways to enhance older adults' social life by drawing on the knowledge and experience gained from two 5‐year‐long research projects (Life 2.0 and WorthPlay) aimed at designing interactive technologies for the elderly	390 people participated in either one or both projects. Participants were aged 55–81 (mean age = 68). The majority had primary‐level education	Understanding the needs, lived experiences and problems of older adults Thinking about and developing possible solutions or services Testing solutions or services Creating a user network	**Life 2.0** Discussion Focus groups Diaries Interviews Persona‐scenarios Storytelling Sketching sessions **WorthPlay** Discussions A game about neighbourhood memories A geo‐located quiz about a book A trivia quiz about Spanish folk parties A game testing general knowledge A literacy game	The study showed how the initial design concept evolved over time as a result of the appropriation of the technologies developed. The findings also challenged the ways in which one might be tempted to think about older people in Human‐Computer Interaction (HCI) and showed the role of community in conducting effective HCI research and design with older people
Valaitis et al. (2019) (Canada)^34^	Using the persona‐scenario method to engage potential end users and other stakeholders in the design of a complex primary health‐care intervention (Health TAPESTRY) for older adults and providers	70 participants: patients (*n* = 15), health‐care providers or community care providers (*n* = 29), community service providers (*n* = 12), and volunteers (*n* = 14)	Understanding the needs, lived experiences and problems of older adults Thinking about and developing possible solutions or services Testing solutions or services	Persona‐scenarios	The persona‐scenario method resulted in the creation of over 400 novel ideas, many of which were integrated into the design of the Health TAPESTRY program
Wherton et al. (2012) (UK)^35^	The ATHENE project, aimed at understanding the health‐care, social care and socio‐cultural needs of older adults, and at exploring how to directly involve older adults and their families in the co‐production of Assisted Living Technologies	28 patients (age range = 60–98) with a wide range of health conditions, social care needs, socio‐economic status, cultural status and family settings	Understanding the needs, lived experiences and problems of older adults Thinking about and developing possible solutions or services	Interviews Life scrapbook	The cultural probe method supported home visit interviews in order to inform them about introducing Assisted Living Technologies such as telecare and telehealth into the homes of older adults

Data were also gathered on when the older adults were involved during the co‐design process. Table 2 presents the level of collaboration of older adults at each stage of the co‐design process according to Brown's Design Thinking framework.^12^ The levels of collaboration of older adults fell into three categories: (1) With; (2) Together; and (3) Without (see legend in Table 2). In short, the levels ‘With’ and ‘Together’ both involve the collaboration of older adult participants, while ‘Without’ involved other stakeholders only. Older adult participants almost always collaborated in the stages of empathising and defining, and ideating. The stages of prototyping and testing were the least documented, and when they were, older adult participants collaborated in less than half.

**TABLE 2:** Level of collaboration of older adults at each stage of co‐design as suggested by Brown's design thinking framework.^12^

Authors	Empathising and defining	Ideating	Prototyping	Testing
Kelemen et al.^30^	With	With	Not mentioned	Not mentioned
Fang et al.^28^	With	With	With	Not mentioned
Valaitis et al.^34^	Without	Together	Together	Not mentioned
Brett et al.^25^	With	With	Not mentioned	Not mentioned
Hoffman et al.^29^	Together	Together	Together	Together
Wherton et al.^35^	Together	With	Not mentioned	Not mentioned
Leask et al.^31^	Together	Together	Together	Not mentioned
Punnaraj et al.^33^	Together	Together	Together	Not mentioned
Dorant and Krieger^27^, ^a^	Without	Not applicable	Not applicable	Not applicable
O'Brien et al.^32^	Without	Together	Together	Together
Righi et al.^24^	Together	Together	Together	Together
Brookfield et al.^26^, ^b^	Not applicable	Not applicable	Not applicable	With
Lu et al.^23^	Together	Together	Not mentioned	Not mentioned

*Note*: ‘With’ represents stages that were performed with older adults' participation. ‘Without’ represents stages that were performed by the research team and other stakeholders without older adults' participation. ‘Together’ represents stages where, depending on the activity, older adults and other stakeholders might have collaborated at different times. For example, a study could have used two co‐design activities at its ideating stage. The first activity might have involved both older adults and other stakeholders, while the second activity might have only included other stakeholders. These two activities then fall under the level of collaboration known as ‘Together’ since they have both been used at the stage of ideating.

^a^ At the time of publication, this study was in its initial phase and only the ‘Empathising and defining’ co‐design stage had been completed. This explains why the other three stages are marked ‘Not applicable’.

^b^ The objective of this paper was to critically assess eight activities. Older adults' collaboration was only required to test the activities and provide feedback on them. Therefore, the first three stages are marked ‘Not applicable’.

### 3.2 Co‐design activities

**FIGURE 2:**
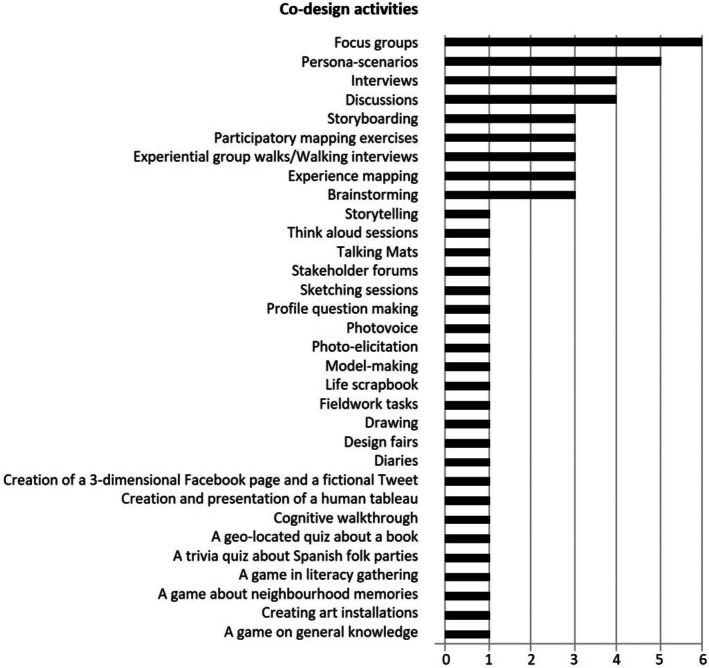
Frequency of occurrence of the co‐design activities across the included studies.

The articles analysed revealed a total of 33 different co‐design activities, not counting the activities that repeated themselves across the studies. The number of activities mentioned per article ranged from one to 12, for an average of about four different activities per article. Persona‐scenarios (*n* = 5^23^, ^24^, ^29^, ^32^, ^34^), focus groups (*n* = 6^24^, ^25^, ^26^, ^27^, ^29^, ^33^), individual interviews (*n* = 4^24^, ^29^, ^33^, ^35^) and discussions (*n* = 4^23^, ^24^, ^27^, ^31^) were the most frequently cited activities. Other activities were single examples, such as creating a human tableau^30^ and think‐aloud sessions.^29^ One of the studies mentioned activities that had never been undertaken before, as they were crafted by the participants and the research team. These included the neighbourhood memories game, the trivia quiz about a book and the quiz about Spanish folk parties.^24^ Detailed descriptions of the activities are provided in Appendix S2. Figure 2 shows the frequency of occurrence of each activity across the included studies.

Most of the activities took place during workshops or events (*n* = 43), while the others were stand‐alone activities (*n* = 10), counting the activities that repeated themselves. The duration of the activities was specified in less than half of the studies and ranged from 1 to 4 h, generally lasting between 1 and 2 h. The stakeholder forum^25^ was the longest activity, and the only one lasting 4 h (including a 1‐h lunch break). Other activities, such as the Life Scrapbook^35^ and the diaries,^24^ were organised differently. Each participant kept a Life Scrapbook or a diary that they could work on for about a week. Data on compensation for the participants were also extracted. Eleven of the 13 studies did not mention if participants were compensated; one study specified that they were not.^30^ Another mentioned that taxi vouchers or parking permits were given to the participants, if needed.^25^

### 3.3 Co‐design activities involved at each stage of the Design Thinking process

**FIGURE 3:**
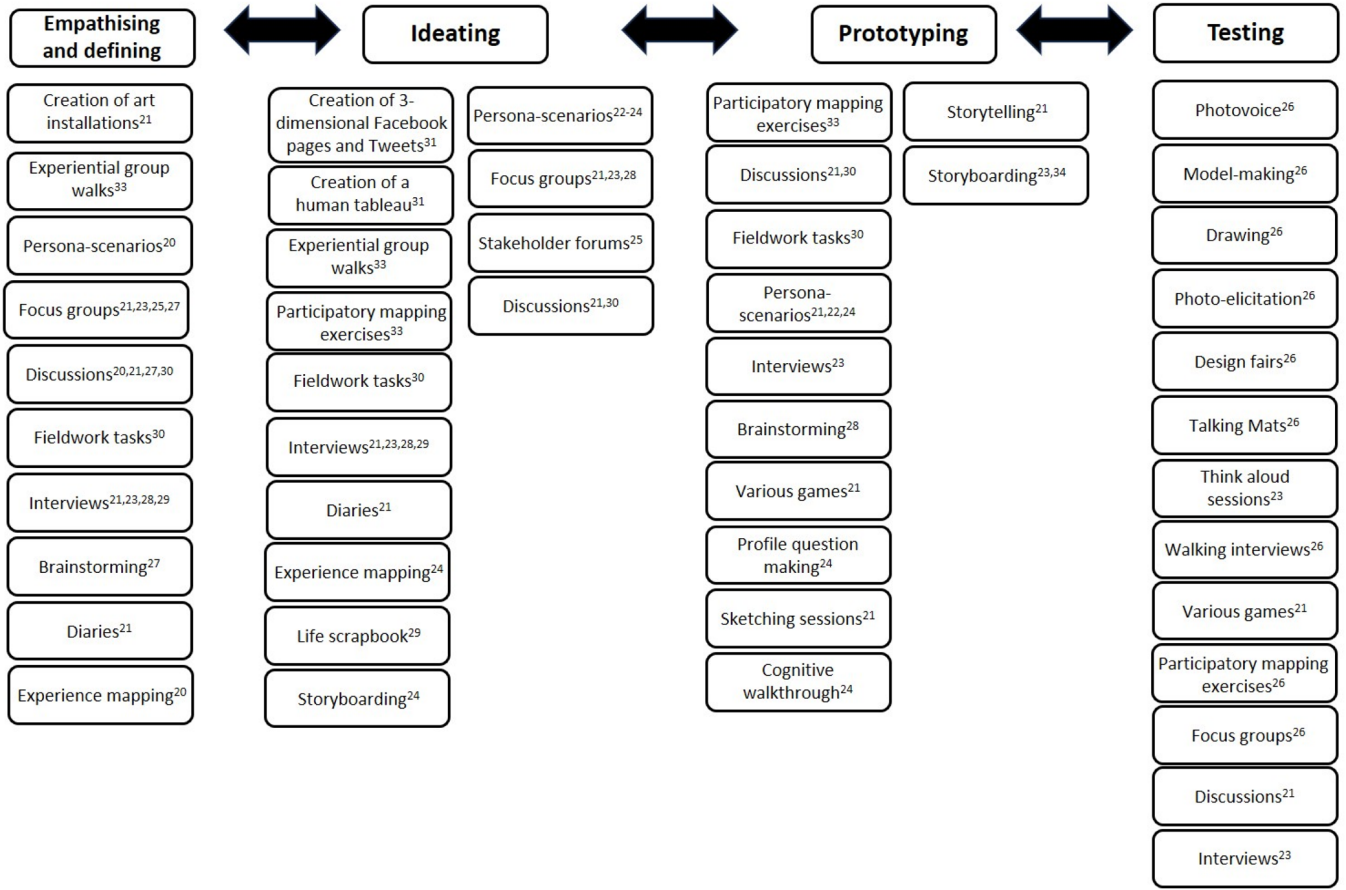
Activities carried out during each stage of co‐design as suggested by Brown's design thinking framework.^12^

This paper was also intended to explore which activities were involved at each of the co‐design stages, as suggested by Brown's Design Thinking framework.^12^ Figure 3 shows the different activities carried out during each stage of the co‐design process: empathising and defining (*n* = 10), ideating (*n* = 14), prototyping (*n* = 12) and testing (*n* = 13). Discussions and individual interviews were each used by at least one study at every step of the co‐design process.

## 4 DISCUSSION

This rapid review synthesises the methods for co‐designing health innovations with and for older adults. More precisely, it describes the purposes of using co‐design processes, shows how and when older adults collaborated in the process and maps the activities used.

Results suggest that co‐design processes can be used for a broad range of purposes. All the studies included in this review sought to understand the needs, lived experiences and problems of older adults. Perhaps, this is indicative of its importance, and it could be considered foundational since other purposes, if applicable, could be built on the knowledge collected. Understanding the needs and lived experiences of older adults is also central to conducting health research projects with HCD methods.

The centrality of collaboration in co‐design supports the transversal participation of older adults in the planning and development of innovations for them. However, as seen in Table 2, some of the studies did not involve older adults at all stages. In fact, some of the stages were carried out by the researchers and other stakeholders. Older adults' participation was the least documented at the prototyping and testing stages, and more detailed for empathising, defining and ideating. One explanation for the lack of information on the participation of older adults in prototyping and testing may be the fact that certain studies were still underway and had only produced preliminary data. It may also be that older adults were absent during design because of disengagement along the co‐design process. Many factors can influence older adults' engagement in a co‐design process, such as the loss of interest of participants, the lack of clarity in the instructions given during the activities, various mental and physical ailments, the maladaptation of the activities to such ailments, not having attendance facilitated (transportation and associated costs) and language barriers.^22^, ^36^ Thus, to ensure full participation of older adults along the co‐design process, they should be involved as early as possible, that is, during the planning of the study, to ensure that the co‐design activities will correspond to their needs and specific context. Finally, although most authors specified whether older adults were engaged in the different co‐design stages, the actual degree of involvement (how and how much older adults were involved) was often not mentioned. The scarcity of such data precludes evaluating the actual impact of older adults' involvement in the co‐design process and may raise questions as to the presence of tokenism, defined as ‘making perfunctory or symbolic efforts to engage communities or patients’.^37^ Not including older adults at all stages of the co‐design process could produce an innovation that is not adapted to their diverse realities and needs. Furthermore, not properly collaborating with older adults in the design process could increase the risks of basing design decisions on ageism or other societal stereotypes.^38^ However, involvement of older adults at all stages of the process and reflecting on the possible contribution of biases and stereotypes does not solve all issues. It cannot be assumed that a better understanding of potential stakeholders will translate automatically into better design decisions.^39^ In addition, the Co‐Constitution of Ageing and Technology (CAT) model^38^ points out that ‘needs and requirements are not stable or fixed “entities”’ but that they are subject to change as people or health innovations and technologies evolve.

The included studies demonstrated that co‐design activities often involve creativity. Among the creativity‐based activities reported were the creation of art installations^30^ and photo‐elicitation.^26^ Some studies opted for more common health research methods that seek an in‐depth individual perspective, such as interviews and focus groups. It can be argued that these techniques are less compatible with the principles of co‐design, in which creativity plays a major role.^8^ However, such forms of investigation may prove necessary or simply appropriate according to the type of data that are sought. This can depend on the nature and the depth of information researchers are looking for (personal opinions or information vs. formulating a problem or generating ideas for innovation), as well as the co‐design stage of the study involved. The choice of more common research methods might have been made because researchers were more familiar with them or simply because they had found that their participants were responding particularly well. Regardless of the presence or absence of creativity in the activities, what might be more important is making an effort to find out how older adults might have felt about their participation in the activities. Making them feel seen and heard, while giving them a space, within a collaborative context, to tell the research teams and other stakeholders about their lived experiences could be a lever in better understanding their needs and requirements than the nature of the activity.

The greatest number of different activities was used at the stages of ideating, prototyping and testing. This could indicate that these stages require numerous activities, and thus, a lot of collaboration and time from the participants.

The findings of this rapid review regarding the diversity and the number of activities carried out in the development of health innovations with older adults is consistent with the findings of a recent systematic review focusing on the co‐design of health information technologies^17^ that highlights the numerous workshop activity options implemented by designers and researchers. Another systematic review, also focussing on the co‐design of technologies for older adults, found that using a combination of activities (both creativity‐based and others that seek in‐depth individual perspectives) increases opportunities for participants' contributions.^16^

### 4.1 Future research

One avenue for future research is to evaluate the added value of co‐design processes in health research and potentially support their use. In fact, we found no comparative studies on the topic. More precisely, it would be relevant to seek out the potential benefits, or perhaps the disadvantages of using a co‐design process, and how they might influence the outcomes of the studies. Similarly, the added value (if any) of introducing more creative activities within co‐design processes in health research (compared to focus groups and individual interviews) also merits future research to support the choice of certain methods over others. More standardised reporting on the application of co‐design processes by experts in innovation, design and health could improve transparency and reproducibility.

### 4.2 Strengths and limitations

One of the strengths of this rapid review is the delivery of rigorous data produced by a limited in‐depth research process. However, the shorter duration of a rapid review increases the risk of selection bias and of overlooking important information, as it is extremely specific. The research strategy, study selection process and screening process were all done by three of the co‐authors. As recommended by Garrity et al.,^20^ a single reviewer extracted the data, but a second reviewer checked for correctness and completeness of extracted data.

Although Brown's Design Thinking framework^12^ was helpful in organising the data collected in this rapid review, the challenge of using a conceptual framework is that it forces co‐design activities that are not completely mutually exclusive into distinct boxes. Finally, although this is a recommended approach when conducting rapid reviews, keyword searches were run in a limited number of databases and did not cover the entirety of the literature. Nonetheless, these three databases were selected based on the stated research questions and recommendations offered by a university librarian.

## 5 CONCLUSIONS

The objective of this rapid review was to synthesise the methods used in health research for co‐designing innovations with and for older adults. This review reported on 33 different co‐design activities. Discussions and individual interviews were the most frequently reported activities and were used at every stage of the co‐design process. The level of collaboration of older adults was more documented at the stages of empathising, defining and ideating. With increased use of co‐design processes, more data will become available, and future literature reviews may enable the development of guidelines for the use of co‐design with and for older adults. Our findings will be of interest to researchers currently using or contemplating the use of co‐design methods with this population by providing ideas and leads on research design and objectives. Ultimately, health and design scientists should work towards creating a true involvement of older adults by addressing issues that may cause the disengagement of older adults in the co‐design process.

## FUNDING INFORMATION

*Fonds de recherche du Québec*—*Santé*, Living Lab platform [grant no 279512, 2020–2023]. Samuel Turcotte is a postdoctoral fellow from the Canadian Strategy for Patient‐Oriented Research and the Canadian Institutes of Health Research (SPOR‐CIHR) (POR Award—Transition to Leadership Stream, 2020–2023). Roxane DeBroux Leduc holds a scholarship award from the *Fonds de recherche du Québec*—*Santé and Fonds de recherche du Québec*—*Société et culture*. Nathalie Bier currently holds a junior 2 Research Scholar Award from the *Fonds de recherche du Québec‐Santé*.

## CONFLICT OF INTEREST STATEMENT

No conflicts of interest declared.

## Supporting information


Appendices S1–S2

## Data Availability

Data sharing is not applicable to this article as no new data were created or analyzed in this study.
