# Supplementary material for: Methods for co‐designing health innovations with older adults: A rapid review
Source: Australas J Ageing. 2025 Jul 16;44(3):e70065. doi: 10.1111/ajag.70065 (PMC12265874; doi:10.1111/ajag.70065)
Supplement: Supplementary file 1 — Appendices S1–S2 [file AJAG-44-0-s001.docx]

**Appendix S1:** Database Search terms

(Co-design OR Codesign OR “Co design” OR Co-creat* OR Cocreat* OR “Co creat*” OR Co-operat* OR Cooperat* OR ”Co operat*” OR Co-produc* OR Coproduc* OR “Co produc*”) AND (Health* OR Care*) AND (Elder* OR Senior* OR “Late life” OR Geriatric* OR Gerontology OR “Nursing home resident*” OR Ageing OR Ageing OR Retire* OR “Older adult”) AND (Protocol* OR Workshop* OR Activit* OR Setting* OR Framework* OR Guideline* OR Guidance OR Step*).

**Appendix S2:** Descriptions of co-design activities

| **Co-design activities**  (articles) | **Descriptions** |
| --- | --- |
| **A game about neighbourhood memories**  **Stages of co-design**: Prototyping, Testing | This game, created by the participants, is about their memories of their neighbourhood. The game made it possible to see how older adults can be involved in creating a future digital version of this game.^24^ |
| **A game in literacy gathering**  **Stages of co-design**: Prototyping, Testing | The participants created a geo-located quiz about the life and works of the Catalan writer Bernat Metge.^24^ |
| **A game on general knowledge**  **Stages of co-design**: Prototyping, Testing | In this game, four quizzes on different topics were created by the participants.^24^ |
| **A trivia quiz about Spanish folk parties**  **Stages of co-design**: Prototyping, Testing | This game intended to explore the idea of conducting simple and playful activities with non-gamers by addressing a topic that is well known to the participants. It was developed by the participants themselves.^24^ |
| **A geo-located quiz about a book**  **Stages of co-design**: Prototyping, Testing | The game was created and played by the participants, who were also members of a book club. This activity showed the degree to which older adults can be interested in participating in a physical and playful activity combining mobile devices and geo-localized technologies.^24^ |
| **Brainstorming**  **Stages of co-design**: Empathising and defining, Prototyping | The participants (who were healthcare professionals) had to use the brainstorming method to reflect on the daily challenges they faced when providing family caregiver support. They converted their ideas into key terms and combined them in a word cloud which they presented to the research team.^27^  Participants were divided into groups and were asked to brainstorm ideas for the community capacity care model for older adults.^33^ |
| **Cognitive walkthrough**  **Stages of co-design**: Prototyping | The participants were asked to test the functionality, usability and aesthetics of the web intervention prototype using a cognitive walkthrough activity. They were given a tablet on which they had to use the intervention. Any feedback provided or technical difficulties reported by the participants were recorded by the facilitators on printed screenshots of each page of the intervention.^32^ |
| **Creation and presentation of a human tableau**  **Stages of co-design**: Ideating | Participants were asked to create a human tableau (a sort of theatrical performance) of the results of the activities and to pretend that they were presenting it to government officials (played by other groups of participants).^30^ |
| **Creation of art installations**  **Stages of co-design**: Empathising and defining | The participants created art installations that represented what was, for them, a healthy community. They were encouraged to use the objects around them (art supplies) to convey what a healthy community looks like for them.^30^ |
| **Creation of a three-dimensional Facebook page and a fictional Tweet**  **Stages of co-design**: Ideating | The participants were asked to use social media as if they were showcasing the results of their activities to the public. They created a three-dimensional Facebook page on a large piece of paper using art supplies. They were also asked to compose a short Tweet about the results.^30^ |
| **Design fairs**  **Stages of co-design**: Testing | At these events, design students displayed models and drawings of age-friendly home and neighbourhood designs that they had created on the basis of issues and rough designs identified and co-created with older adults in prior activities. The older adult participants present at these events moved between the displays, talked to students about their prototypes and suggested how to further improve their age-friendliness.^26^ |
| **Diaries**  **Stages of co-design**: Empathising and defining, Ideating | The participants had to keep a diary to provide a brief description of their everyday activities conducted in or outside their home, with or without the use of technologies, and with or without friends or family.^24^ |
| **Discussions**  **Stages of co-design**: Empathising and defining, Ideating, Prototyping, Testing | In Dorant & Krieger,^27^ discussions took place in small groups of three or four professionals and focused on the goals and expectations of the family caregiver support concept that would eventually be created.  In their study, Leask et al^31^ carried out multiple workshops during which the main activity was simply discussing various fieldwork tasks (see next activity) the participants had to engage in regarding the co-creation of a public health intervention to reduce sedentary behaviour in older adults.  Lu et al^23^ does not elaborate on the content of the discussions.  In Righi et al,^24^ discussions were used on multiple occasions. Sometimes participants had informal conversations about the observations they made during an activity or while they played games. They also had debriefing sessions when elements gathered during activities were validated by the participants. |
| **Drawing**  **Stages of co-design**: Testing | Design students and older adult participants sketched out their impressions of their neighbourhood by identifying what they liked and disliked about it, which elicited a positive or negative emotional response in them.^26^ |
| **Experience mapping**  **Stages of co-design**: Empathising and defining, Ideating | Lu et al^23^ do not elaborate on experience mapping, but mention that the ‘maps’ were used to demonstrate the daily activities of the older adult participants.  This activity consisted of experience mapping of transitioning to retirement, and it was aimed at mapping different retirement pathways by considering key factors such as the most appropriate time to retire, when retirement is most accepted, effective transition and the stages of retirement. This allowed participants to share their own experiences and discuss how people feel once they retire. The participants were also asked to provide ideas for potential interventions to help people face the challenges of transitioning to retirement.^32^ |
| **Experiential group** **walks**^a^  **Walking interviews**  **Stages of co-design**: Empathising and defining, Ideating, Testing | Participants and researchers went on group walks in participants’ local neighbourhoods and discussed their impressions of their environment. The walks were meant to facilitate the identification of existing facilities and resources in the community, the identification of opportunities for social activities, the potential facilitators and barriers to moving around the community, while also giving the participants a chance to express their feelings about the location and enabling social participation. Both studies carried out this activity in a very similar manner.^26,28^ |
| **Fieldwork tasks**  **Stages of co-design**: Empathising and defining, Ideating, Prototyping | Between workshops, participants had to complete a series of tasks to help develop the public health intervention. Examples of these tasks are gathering and printing media images of sitting positions in different contexts and identifying the context of prolonged sitting periods, as well as strategies to break up sedentary behaviour.^31^ |
| **Focus groups**  **Stages of co-design**: Empathising and defining, Ideating, Testing | Brett et al^25^ managed focus groups prior to conducting their stakeholder forums. The focus groups were aimed at aligning researcher and participant goals, and at ensuring that the goals reflected participants’ needs. It also established stakeholder forum core themes.  Brookfield et al^26^ do not explain how the focus groups were managed.  In Dorant & Krieger,^27^ focus groups were created with the participants (who were healthcare professionals) to discuss the following themes: caregivers’ needs, information, expertise, skills, management, mandates, etc.  Hoffman et al^29^ performed a series of focus groups with community members, clinical experts, counsellors and policy-makers to produce a preliminary list of potential decision support needs for the health intervention.  Punnaraj et al^33^ conducted focus groups in which a facilitator asked the participants a series of questions. The questions were specific to the participants, as groups were divided according to their occupation (e.g. healthcare professionals, older adults and their family members and healthcare volunteers). The questions assessed the needs of older adults and their families, and the services available from different healthcare providers. The focus groups helped researchers determine whether a community capacity model was appropriate for the community.  Focus groups in Righi et al^24^ were aimed at having participants share life stories and daily habits. |
| **Interviews**  **Stages of co-design**: Empathising and defining, Ideating, Prototyping, Testing | In Hoffman et al, ^29^ participants were interviewed (interviews were semi-structured) following the completion of the storyboarding and Think Aloud activities. They were asked to provide suggestions for improving the website.  The facilitator conducted in-depth interviews with all 140 elderly participants. These served to gather data on the needs of older adults in the community. The facilitator assessed the physical abilities, social and economic status, and their relationship with their families.^33^  The local social services director was interviewed by the researchers to get an idea of the public services available to older adults and identify potential issues regarding these services.^24^  Each participant received three home visits. On the first visit, the facilitator explained the purpose of the project. On the second visit, the facilitator conducted semi-structured interviews that focused on senior participants’ routines, health, social networks and technology use. On the third visit, the facilitator interviewed the participants about their scrapbooks (see next activity) and conducted a home tour. The home tours sparked further discussion about specific areas of the home, mobility issues, problems with space arrangement and so on.^35^ |
| **Life scrapbook**  **Stages of co-design**: Ideating | Participants were given the Home and Life Scrapbook, a booklet containing seven activities serving to collect information on physical, emotional, social and environmental factors related to health and independence at home. Along with the scrapbook, participants received a digital camera and a guide on how to use the materials. Examples of participant activities in the booklet are drawing relationships with people, places and objects, stating three things they would like to improve or change about their lives, drawing the outline of a body and using it to mark symptoms or impairments, and journaling about activities and events.^35^ |
| **Model-making**  **Stages of co-design**: Testing | In advance of this activity, design students made a simple 3D base structure from modelling foam to represent the participants’ neighbourhood. During the activity, the participants (with the help of the design students) revised the model by removing elements they did not like, introduced new structures and changed the environment. In another instance, the participants worked with older adults to create a prototype of the older adults’ ideal home that would respond to their various needs.^26^ |
| **Participatory mapping exercises**  **Stages of co-design**: Ideating, Prototyping, Testing | These exercises involved the co-production of a spatial map presenting the local environment and its resources. Maps could be created digitally, from a blank sheet of paper or by annotating an existing map. Essentially, this process tries to capture the ‘sense-of-place’ experienced by the participants. This concept refers to the place of identity, sense of purpose, belonging and living a meaningful life within one’s community.^40^ The maps can include or highlight facilities and resources present in the community, barriers to moving around, the participants’ feelings about the locations, visual data, participants’ personal narratives, gaps in community service delivery, ideas for future community space design, comments about the areas the participants like or dislike and why, etc. Both studies carried out this activity in a very similar manner.^26,28^ |
| **Persona-scenarios**  **Stages of co-design**: Empathising and defining, Ideating, Prototyping | In persona-scenario activities, participants either work together to create a potential end user (a persona) engaged in a scenario or are assigned a pre-existing persona-scenario in which the persona must navigate certain challenges. The characteristics of the persona are usually representative of the participants. For example, a persona could be a 59-year-old female. A scenario is a short story that communicates information from which to draw requirements. For example, this 59-year-old female is about to retire. The participants have to work through the possible challenges of the situation. Hoffman et al,^29^ O’Brien et al, ^32^ and Valaitis et al^34^ used the same principles to guide their persona-scenario activities.  Lu et al^23^ and Righi et al^24^ provided very little detail about how they performed the activity. |
| **Photo-elicitation**  **Stages of co-design**: Testing | This activity involves introducing images during an interview to spark reflections and discussion. In one of the workshops, the participants were shown six researcher-generated photographs of various outdoor environments. Half the images contained features that, according to previous research, are viewed favourably by older adults, and the other half had features that are viewed negatively. The questions asked throughout the interview were intended to elicit the participants’ perceptions and attitudes toward these features.^26^ |
| **Photovoice**  **Stages of co-design**: Testing | In this activity, the participants use photographs to record some aspects of their lives, such as the needs and assets of their neighbourhoods or positive and negative attributes of certain locations. The photographs and their meaning are then discussed and critically reflected upon during one-on-one conversations or in a group setting.^26^ |
| **Profile question making**  **Stages of co-design**: Prototyping | The participants were asked to come up with questions to further personalise the web intervention. The questions were meant to inform the web intervention about the person’s attitudes and habits in relation to their lifestyle in order to adapt it to meet the goals and needs of the user. Participants wrote the question examples on cards and then discussed them one by one.^32^ |
| **Sketching sessions**  **Stages of co-design**: Prototyping | Some participants engaged in sketching sessions where they discussed and sketched the design of the service web platform.^24^ |
| **Stakeholder forums**  **Stages of co-design**: Ideating | Stakeholder forums were conducted with participants and researchers and consisted of social activities providing an opportunity to meet and get to know each other, give an overview of the project and discuss specific topics.^25^ |
| **Talking Mats**  **Stages of co-design**: Testing | Talking mats is a picture-based method that helps individuals with communication difficulties to express their point of view. It involves participants looking at a picture representing an activity, an item, a relationship and so on, and indicating their opinion about it by positioning it along a visual scale that captures some concept of interest, such as preference. The research team developed a visual scale of importance, going from not important to important. The pictures illustrated 17 features of the home and of outdoor environments that were identified by the World Health Organization as necessary components of age-friendly homes and cities. The participants had to place them on the scale according to their opinions.^26^ |
| **Think aloud sessions**  **Stages of co-design**: Testing | Participants were asked to ‘think aloud’ (speak out loud) while using the website. This informed researchers about the opinions, difficulties, questions and ideas the participants had as they were trying out the website.^29^ |
| **Storyboarding**  **Stages of co-design**: Ideating, Prototyping | Participants were given paper prototypes of the website on which they could draw, annotate and give feedback on the text, layout, graphical elements, questions and content of the website.^29^  Storyboarding allowed the participants to put together their ideas for new interventions aimed at supporting ideal retirement experiences. Each group provided an outline of a potential intervention. This outline included the name of the intervention, how it would be advertised, its features and the behaviours it would help to promote. ^32^ |
| **Storytelling**  **Stages of co-design**: Prototyping | The participants had to come up with different ideas for future services to enhance older adults’ social life. Some of the ideas were then selected and presented to the entire group of participants through storytelling. Participants were invited to indicate positive and negative aspects of the services suggested, which were collaboratively written down on Post-its.^24^ |

**^a^** The same activity is named differently in the two studies.

**References**

40. Kyle G, Chick G. The social construction of a sense of place. Leis Sci. 2007;29(3):209-225. doi:10.1080/01490400701257922
